# Relationship between brain plasticity, learning and foraging performance in honey bees

**DOI:** 10.1371/journal.pone.0196749

**Published:** 2018-04-30

**Authors:** Amélie Cabirol, Alex J. Cope, Andrew B. Barron, Jean-Marc Devaud

**Affiliations:** 1 Department of Biological Sciences, Macquarie University, North Ryde, NSW, Australia; 2 Research Center on Animal Cognition, Center for Integrative Biology, Toulouse University, CNRS, UPS, Toulouse, France; 3 Department of Computer Science, University of Sheffield, Sheffield, South Yorkshire, United Kingdom; University of North Carolina at Greensboro, UNITED STATES

## Abstract

Brain structure and learning capacities both vary with experience, but the mechanistic link between them is unclear. Here, we investigated whether experience-dependent variability in learning performance can be explained by neuroplasticity in foraging honey bees. The mushroom bodies (MBs) are a brain center necessary for ambiguous olfactory learning tasks such as reversal learning. Using radio frequency identification technology, we assessed the effects of natural variation in foraging activity, and the age when first foraging, on both performance in reversal learning and on synaptic connectivity in the MBs. We found that reversal learning performance improved at foraging onset and could decline with greater foraging experience. If bees started foraging before the normal age, as a result of a stress applied to the colony, the decline in learning performance with foraging experience was more severe. Analyses of brain structure in the same bees showed that the total number of synaptic boutons at the MB input decreased when bees started foraging, and then increased with greater foraging intensity. At foraging onset MB structure is therefore optimized for bees to update learned information, but optimization of MB connectivity deteriorates with foraging effort. In a computational model of the MBs sparser coding of information at the MB input improved reversal learning performance. We propose, therefore, a plausible mechanistic relationship between experience, neuroplasticity, and cognitive performance in a natural and ecological context.

## Introduction

A central tenet of contemporary behavioural neuroscience is that there is a bidirectional relationship between experience and brain structure. Experience and learning change brain structure by neuroplasticity, and structural changes to the brain have consequences for information processing and thereby further experience and learning [1–4]. Several classic studies have contributed evidence to this tenet [5–9], but few studies have shown both how experience changes brain microstructure and the consequences of these changes for cognitive function [2].

Experience-dependent changes in neuroanatomy have been documented in birds and mammals (including humans) in relation to exploring new environments or developing new behavioural capacities [7,8,10–13]. Similar changes have also been seen in short-lived insects [5,6,14–16]. There is now no doubt that experience-dependent network changes support new memories [4,17], but it is less clear how such changes might support learning of new skills or new cognitive abilities. Ethological examples of a relationship between neuroplasticity and cognitive performance are rare [2]. Here we used the natural behaviour and ecology of the honey bee to examine both aspects of the relationship between brain and experience. Honey bees have provided an important natural example of experience-dependent neuroplasticity, as foraging behaviour is associated with marked changes in brain structure [18–20].

Honey bees begin adult life working inside the hive, but typically when more than 14 days old as adults they transition into a foraging role [21]. The onset of foraging exposes bees to new environments and places demands on bee cognition for spatial navigation, and identifying profitable sources of nectar or pollen, in an ever-changing environment [22,23]. The onset of foraging is preceded by a series of orientation flights in which bees learn the hive location [21,24]. These behavioural changes are accompanied by changes in the mushroom bodies (MBs) [5,18–20,25], which are regions of the bee brain needed for certain learning tasks [26,27]. Foragers have larger MBs than nurse bees that work inside the hive [5,18], and the MBs continue to increase in size with additional foraging experience [18,19]. The experience-dependent growth of the MBs is caused by dendritic arborisation [18,20,25] in their input subregions; the lips and collars of the MB calyx which receive olfactory and visual inputs respectively [28]. In both subregions axon terminals of input neurons connect to the dendrites of intrinsic MB neurons, thus forming synaptic boutons (also called microglomeruli). Despite the growth in volume and dendritic arbours, foragers have fewer synaptic boutons in the lip and collar regions than younger bees working in the hive [25], suggesting a synaptic pruning at either the onset of foraging or during the orientation flights that immediately precede foraging. Such synaptic pruning in the collar has been suggested to be induced by light exposure in *Cataglyphis* ants and honey bees [29,30]. The functional consequences of this experience related structural plasticity of the MBs has been much speculated on [5,18,31], but remains unclear.

Here we examined how the experience-dependent changes in honey bee MB microstructure correlated with performance in a cognitive task which is dependent on MB function: reversal learning [26]. In reversal learning, bees learn first to respond to a rewarded odour A and not to a non-rewarded odour B (A+B-). In a second phase, they learn the reverse contingencies (A-B+). The resolution of this task requires flexibility in learned behaviour [26,32]. This task is expected to be particularly meaningful for foraging bees, as they need to update the value of floral cues (e.g. odorants) as indicators of food, because nectar production varies in time [26,32]. Our results reveal a clear relationship between experience-dependent changes in MB synaptic bouton number and both an increased reversal learning performance at the onset of foraging, and a drop in reversal learning performance in more experienced foragers.

## Materials & methods

Experiments were carried out during the summer of 2016 at Macquarie University (Sydney, Australia). Approximately 1,500 newly emerged adult honey bees (*Apis mellifera*) were collected from frames of emerging brood from three different colonies, including a colony headed by a single inseminated queen for the brain immunohistochemical analyses. Frames were placed in a dark incubator (33°C) for 24 h for bees to emerge.

### Radio frequency identification (RFID) system

Newly emerged bees were equipped with a RFID tag (INVENGO) [33–35] glued to their dorsal thorax with super glue, and marked with a dot of paint on the tag to identify their birth date. Tagged bees were introduced to a hive equipped with an RFID antenna (INVENGO) at the entrance which could detect individual bees’ entries and exits from the colony thanks to the unique 12-byte hexadecimal identifier of each RFID tag. From these we reconstructed foraging trip durations. Trips of < 30s were removed from the data as they were considered to include walking at the entrance or defecation flights. On the day before introducing the newly emerged bees, the hive was displaced to its final position in order to induce some of our focal bees to forage before a normal age, which they do to replace the old foragers that returned to the previous hive location and got lost [36]. There, the hive was connected to the RFID system.

### Reversal learning

When tagged bees were between 22 and 26 days old, some were arbitrarily collected from the hive entrance in the afternoon. Collected bees were briefly immobilized on ice and harnessed in metal tubes allowing movements of the antennae and mouthparts only [27]. They were then fed 15μL of sucrose solution (50% w/w) and kept in darkness, at room temperature overnight. The reversal learning task started on the following morning. Only bees that demonstrated the proboscis extension response (PER) when touching the bee’s antennae with a toothpick soaked in sucrose solution (50% w/w) were used (>95% of collected bees; n = 94).

In the first phase of reversal learning, bees were trained to respond to an odour A rewarded with sucrose, but not to an unrewarded odour B (A+ vs. B-). In the reversal phase, one hour later, bees had to learn the opposite rule (A- vs. B+). Each phase consisted of 5 presentations of each odour (5 trials) in a pseudo-random order, with an inter-trial interval of 8min [26,37]. The conditioning odours were 1-nonanol and heptanal (Sigma-Aldrich). Their use as odour A or B alternated between testing days. During each learning trial of 40s, the bee was placed in front of an odourless airflow passing through an empty syringe for 15s. The odour was then presented by passing the airflow through a syringe containing a filter paper soaked with 4μL of pure odorant for 4s, the last second of which overlapped with a sucrose presentation for 4 seconds. This odour delivery system was automatized. The presence or absence of conditioned response (PER during the odour presentation) was noted as 1 or 0 respectively. Inversion Scores (IS) were then calculated for each bee as the difference between its responses to B+ and A-, for each of the last two trials of the reversal phase. These trials were used to define learners (IS = 1) and non-learners (IS = -1 or 0).

### Immunostaining procedure

Of the conditioned bees, 18 were sampled arbitrarily to analyse MB structure, irrespective of their learning status or foraging activity. Synapsin immunostaining of whole-mount brains was performed following Groh et al [38]. Briefly, brains were dissected and fixed in paraformaldehyde (4% in Phosphate Buffer Saline–PBS—0.01M), rinsed with PBS, permeabilised in PBS-Triton X-100 (Tx) (2% and 0.2% successively), and blocked with 2% normal goat serum (NGS) in 0.2% PBS-Tx. They were then incubated with the α-synapsin primary antibody (SYNORF1; DSHB; 1:10 in 0.2% PBS-Tx—2% NGS) for 4 days, rinsed in PBS and incubated with the secondary antibody (Alexa Fluor 488–conjugated goat anti-mouse; Fisher Scientific; 1:250 in 1% NGS-PBS) for 3 days. After rinsing in PBS, brains were dehydrated in an ascending ethanol series (30%, 50%, 70%, 90%, 95%, 3X 100%, 10min per step). Whole brains were cleared and mounted in methyl salicylate for imaging.

### Image acquisition and analyses

Images of the whole-mount brains were acquired using a laser scanning confocal microscope (LEICA SP5). For volume measurements of the lip and dense collar regions of the MBs, stacks were imaged through the entire right medial calyx with a 5μm interval between optical sections (10x/0.4 objective, digital zoom 3). To quantify synaptic boutons in the same calyx, optical sections were taken at a 0.5μm interval over a depth of 10μm (63x/1.4 objective, digital zoom 2).

Images were processed using the 3D reconstruction software AMIRA 3.0 (FEI Visualization Sciences Group, Düsseldorf, Germany). The boundaries of the lip and dense collar volumes were traced manually for each section and volume reconstructed by interpolation. The numbers of synapsin-positive profiles were counted within cubic sampling volumes (1000μm^3^) located within the lip and dense collar (4 and 3 sampling volumes respectively). Synaptic bouton density was averaged over the sampling volumes for each individual. The absolute number of synaptic boutons per lip and dense collar was obtained by extrapolating the mean density to the measured volume of the brain region.

### Computational model of MB function

We hypothesised that changes in synaptic bouton number could affect connection density between the inputs to the MBs and the intrinsic neurons of the MBs and that this would change how sparsely olfactory information was coded in the MBs. We developed a computational model of the MBs to assist in exploring the theoretical consequences of varying connectivity parameters within the MBs for performance in reversal learning. The model was built upon an abstraction of the MB circuit proposed by Bazhenov et al [39], which provided a now well-established conceptual model for how the MBs can function in olfactory classification and learning.

The main structure of the model consists of an associative network with three neural network layers. Adapting terminology and features from the insect brain, we labelled these: input neurons (IN), a large middle layer of MB intrinsic neurons called Kenyon cells (KC), and a small output population of MB extrinsic neurons (EN). We also considered the GABAergic inhibitory protocerebral tract (PCT) neurons in the model, which provide inhibitory feedback to the KC [40].

To provide inputs from the odorants A and B the IN were divided into two subsets of 16 neurons, one for each odorant. The input values when the odorant is presented were chosen randomly in the range {0.9, 1.1} and fixed for the duration of the experiment. The connection weights between the IN and KC were formed by a fixed matrix, where a connection between the *i*th IN and the *j*th KC is denoted *c*_*ij*_. The probability of an IN and a KC being connected determines how sparse or dense the connectivity is; for a probability of one all neurons are connected, and a probability of zero leads to no connections. We used two values for the probability: sparse (0.15) or dense (0.22), mathematically described by *p*_*IN->KC*_ = {0.15, 0.22}. These values are slightly higher than those used by Bazhenov et al [39] to compensate for the sparsening effect of inhibition by the PCT and therefore maintain the number of active KC for the sparse case. All connections have a fixed strength of one.

Each model KC sums its inputs, subtracts a threshold value *b*, and outputs the final value if it is greater than zero using the Heaviside function *θ*. The value of *b* is chosen to ensure only KC with two or more active inputs produce an output, and therefore is set to a value of 1.4. The connection weights from the KC to the EN are plastic and changed as the model was rewarded and learned, and every KC is connected to every EN. The connection strength between the *j*th KC and the *k*th EN (denoted *w*_*jk*_) can take a value between zero and one. Learning-related plasticity takes place in all synaptic weights according to the equation:

$$\Delta w=\alpha (R-R_{b})\times presynaptic\;\mathrm{with}\;\mathrm{probability}\;p=0.1$$

where *α =* 0.13 is the learning rate of the weights, *R = 1* if reward is given, and zero in all other cases, *R*_*b*_ = 0.62 is a reward baseline. These values were chosen so that the synapse learned at approximately half the rate that it forgets. With these values acquisition rate matched that found in real bees. The term *presynaptic* is 1 if the presynaptic neuron is active and 0 elsewhere. It should be noted that reward was given, and learning occurs, on proboscis extension only. To match the initial condition of the bees in the experimental procedure a single punished trial was used to reduce the response of the model to both odors.

The extrinsic neurons, EN, were modelled as two distinct sub-populations dedicated to triggering proboscis extension (which we shall term Extend) and retraction (termed Retract). In the model, the proboscis is extended if the total output of the Extend sub-population is greater than the total output of the Retract sub-population, as long as the total activity of both sub-populations together is greater than 0.1 (i.e. once a suitable threshold for the decision has been reached). For the inhibitory PCT neurons, the output of the *l*th neuron in this population is described by the variable *s*_*l*_. This inhibition increases the sparseness of active KC by suppressing weakly active neurons below the threshold for activity, leading to fewer KC being active for the same stimulus with PCT inhibition as without [41,42]. As these neurons are fed by all the KC, a high value of 150 for *b*_*s*_ (the threshold for output) was used. A global weighting *w*_*PCT*_ = 1x10^-5^ was used to set the level of inhibition to replicate the performance of experimental control bees This value is low due to the high value of the PCT neurons and is chosen to avoid oscillations in the tight loop between the KC and PCT neurons while still providing a strong inhibitory effect.

Mathematically the model is formulated as follows where *x*_*i*_ is the output of the *i*th IN, *y*_*j*_ is the output of the *j*th KC, *z*_*k*_ is the output of the *k*th EN and *s*_*l*_ is the output of the *l*th PCT neuron. The constant values are as described above.

$$y_{j}=(\sum_{i=0}^{N_{IN}}c_{ij}x_{i}-b-w_{PCT}\sum_{l=0}^{N_{PCT}}s_{l})\theta (\sum_{i=0}^{N_{IN}}c_{ij}x_{i}-b-w_{PCT}\sum_{l=0}^{N_{PCT}}s_{l})$$

$$z_{k}=(\sum_{j=0}^{N_{KC}}w_{jk}y_{j})\;\theta \;\left(\sum_{j=0}^{N_{KC}}w_{jk}y_{j}\right)$$

$$s_{l}=(\sum_{j=0}^{N_{KC}}y_{j}-b_{s})\theta (\sum_{j=0}^{N_{KC}}y_{j}-b_{s})$$

The numbers of neurons in each population were as follows: *N*_*IN*_ *=* 32 is the number of IN; *N*_*KC*_ = 5,000 is the number of KC. There are 6 PCT neurons, and 4 EN in each of the Extend and Retract subsets.

Using the model we examined performance of virtual bees in the reversal learning task. The experimental protocol for the model was identical to that used with real bees. To explore how changing connection properties between the IN and KC might impact on reversal learning performance we modelled three conditions for the virtual bees: sparse connectivity between the IN and the KC, dense connectivity between the IN and the KC, and finally with the inhibitory PCT neurons silenced. For each condition we used a ‘models as animals’ approach. Different random seeds for generating the EN to KC connectivity were used to create a set of 200 virtual bees, and each bee was tested individually.

### Statistical analyses

R 3.2.3 was used for data analyses and graphic representations (R Core Team (2015)) [43]. For reversal learning, the responses to the odours were analyzed using a repeated-measurement ANOVA (the data met the criteria to apply an ANOVA to a dichotomous dependent variable [44]), followed by a Tukey honest significant difference (HSD) *post hoc* analysis to compare response levels to the two odors in the different learning trials within each group. The results of the Tukey HSD analysis are reported in the text for the 5^th^ trial of each phase, which show the ability of bees to learn the rule by the end of the task, and for the 4^th^ trial of the reversal phase as some bees were able to solve the task as soon as in the 4^th^ trial, thus demonstrated a higher performance. Inversion scores and neuroanatomical differences between groups were compared using a Mann-Whitney U test. Spearman ranks correlations were used to assess the relationship between brain structure and foraging intensity.

## Results

RFID data provided the cumulative time spent outside the hive for each bee. Bees were assumed to have begun foraging when they had accumulated > 30 min time outside the hive [24,34]. Bees with < 30 minutes of time outside the hive were considered as performing orientation flights (‘orientating bees’) [24,34]. Bees that began foraging when less than 14 days old as adult were classified as precocious foragers [34]. We were thereby able to compare reversal learning performance of bees with different foraging durations (based on cumulative time foraging), in the whole sample, and also among precocious and normal-age foragers independently.

### Reversal learning performance declines with foraging experience

**Fig 1:**
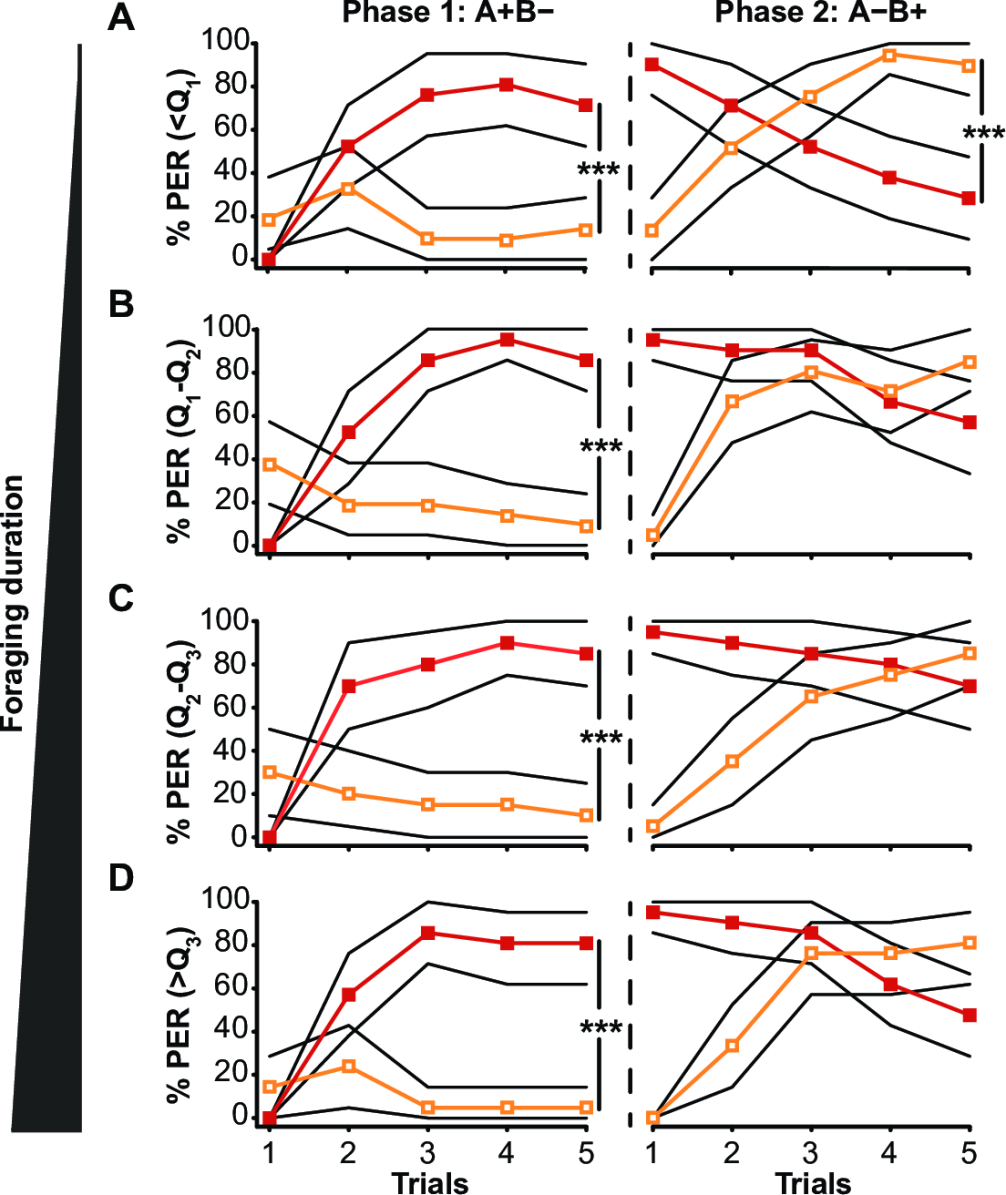
Change in reversal learning performance with duration of foraging. Percentages of individuals displaying PER in response to odours A (*red line*) and B (*orange line*) are shown, during the first phase (A+B-) and the reversal phase (A-B+) of the reversal learning task. Results are presented for bees with increasing foraging durations defined by the 1^st^ quartile (Q_1_ = 113.8min), median (Q_2_ = 381.3min), and 3^rd^ quartile (Q_3_ = 653.5min) of the total amount of time foraging of the whole sample. The bootstrapped 95% confidence intervals are indicated by the black lines. [**(A):** n = 21, **(B):** n = 21, **(C):** n = 20, **(D)**: n = 21] *** p < 0.0001, Tukey HSD *post hoc* analysis.

We first investigated the effect of foraging duration on performance in reversal learning, i.e. excluding orientating bees **(Fig 1)**. For this, our sample was divided into four groups of increasing foraging durations, defined by the 1^st^ quartile (113.8min), the median (381.3min) and the 3^rd^ quartile (653.5min) of the distribution of foraging durations recorded in our complete sample of 83 foragers. Foraging duration clearly affected performance in the reversal phase of the learning task, but not the ability to solve the simple discriminative task of the first phase **(Fig 1)**. Responses to the rewarded odour (A+) and non-rewarded odour (B-) did not differ between the 4 foraging-experience groups in the first learning phase (Repeated-measure ANOVA; *Group* effect: F = 0.58, p = 0.63). They all reached significant discrimination in the last trial (Tukey HSD *post hoc* analysis; p < 0.0001 in all groups). In the reversal phase, however, although all groups changed their response patterns (*Trial x Odorant* interaction: F = 107.10, p < 0.0001), only bees in the first quartile of foraging durations responded more to B+ than to A- by the last learning trial (p < 0.0001; p > 0.40 for the other groups). We infer from this that foraging activity performed beyond a certain duration (corresponding to 113.8 minutes in our conditions) reduced performance in a reversal learning task. This value of 113.8 minutes of foraging was subsequently used as a threshold between ‘short’ and ‘long’ foraging durations in the following analyses.

### Precocious foragers are more affected by the decline in reversal learning performance

**Fig 2:**
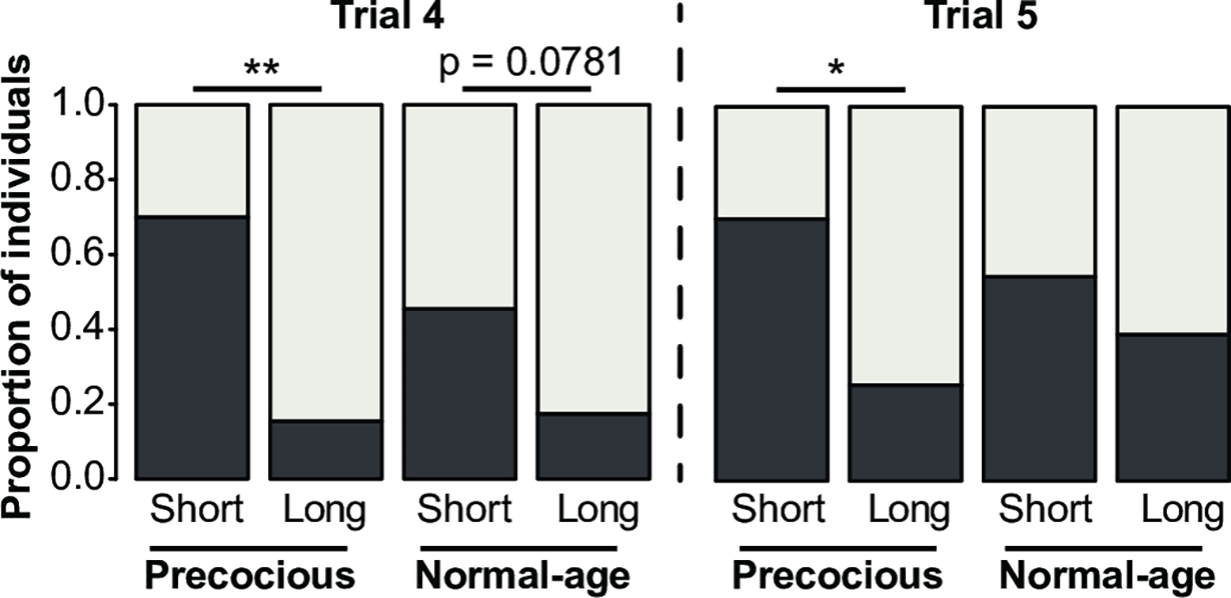
Reversal learning performance of precocious and normal-age foragers with short or long foraging durations. The proportions of non-learners (*NL*: *light grey*) and learners (*L*: *dark grey*) in the last two trials of the reversal phase (trials 4 and 5) are displayed. For each trial, bees were defined as non-learners or learners according to the value of their individual inversion score (see Methods; NL: IS = -1 or 0; L: IS = 1). The IS were compared between precocious and normal-age foragers, with either short or long foraging durations corresponding respectively to durations within or greater than the 1^st^ quartile of the whole sample (113.8min). [*Precocious*: short: n = 10, long: n = 39; *Normal-age*: short: n = 11, long: n = 23] * p < 0.01; ** p < 0.005, Mann-Whitney U-test.

Because our bee population included bees that started foraging with a normal age range or precociously, we compared their learning abilities. While performance of precocious and normal-age foragers remained unaffected by foraging duration in the first learning phase (data not shown; Kruskall-Wallis H-test; *Trial 4*: p = 0.600; *Trial 5*: p = 0.167), the decline of reversal performance with foraging experience was more apparent in precocious foragers **(Fig 2)**. Precocious foragers with a long foraging duration had lower inversion scores (IS) compared to those with a short foraging duration in the last two trials of the reversal phase (Mann-Whitney U-test: *Trial 4*: U = 310.5, p < 0.001; *Trial 5*: U = 286, p < 0.01). This was not the case in normal-age foragers (*Trial 4*: U = 165; p = 0.078; *Trial 5*: U = 146, p = 0.416). This indicates that normal-age foragers are more resistant than precocious foragers to the foraging-related decline in reversal learning capacities.

### Beginning foraging is associated with an improvement in reversal learning abilities

**Fig 3:**
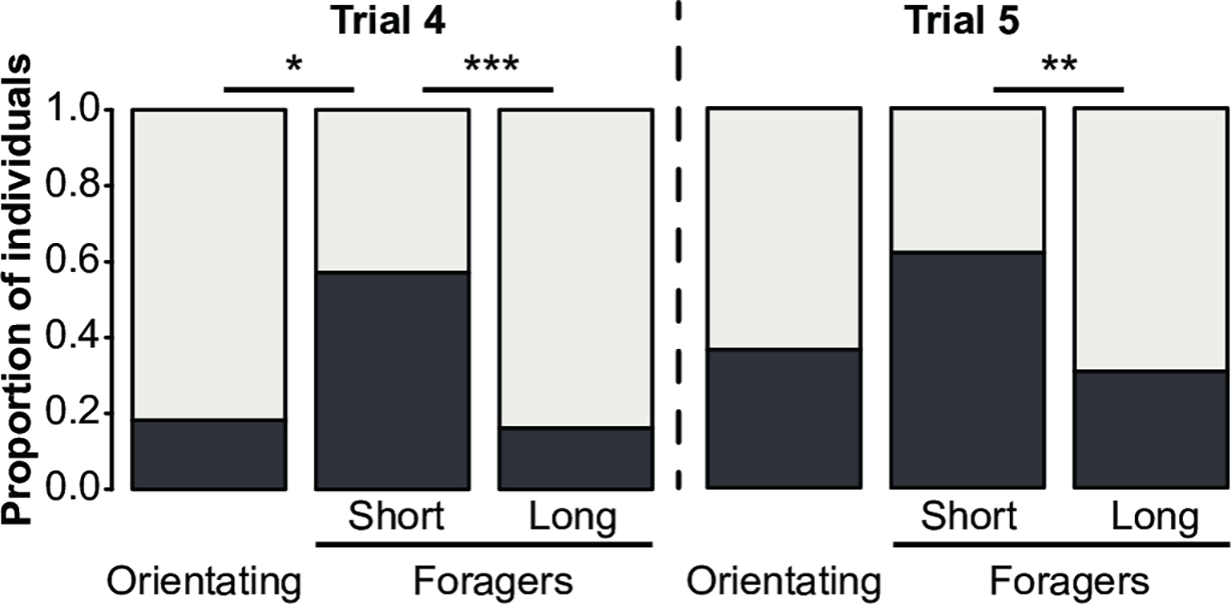
Reversal learning performances of orientating bees and foragers with short or long foraging durations. The proportions of non-learners (*NL*: *light grey*) and learners (*L*: *dark grey*) in the last two trials of the reversal phase (trial 4 and 5) are displayed. For each trial, bees were defined as learners or non-learners according to the value of their individual inversion score (see Methods; NL: IS = -1 or 0; L: IS = 1). The IS are compared between orientating bees and foragers, with either short or long foraging durations corresponding respectively to durations within or outside the 1^st^ quartile of the whole sample (113.8min). [*Orientating*: n = 11; *Foragers-Short*: n = 21; *Foragers-Long*: n = 62] * p < 0.05; ** p < 0.01; *** p < 0.0005, Mann-Whitney U-Test.

The effect of foraging onset on reversal learning was assessed by including the group of orientating bees (total amount of time outside < 30min) in the analysis. We compared their performance with that of foraging bees with short and long foraging durations **(Fig 3)**. The IS differed among the three groups in the last two trials of the reversal phase (Kruskall-Wallis H-test; Trial 4: p < 0.001; Trial 5: p < 0.05). More precisely, beginning foraging was associated with an increase in reversal learning performance, as bees with short foraging duration had a higher IS in the 4^th^ trial than orientating bees (Trial 4: U = 61.5, p < 0.05; Trial 5: U = 149, p = 0.135).

### Structure of the MBs varies with foraging onset and experience

**Fig 4:**
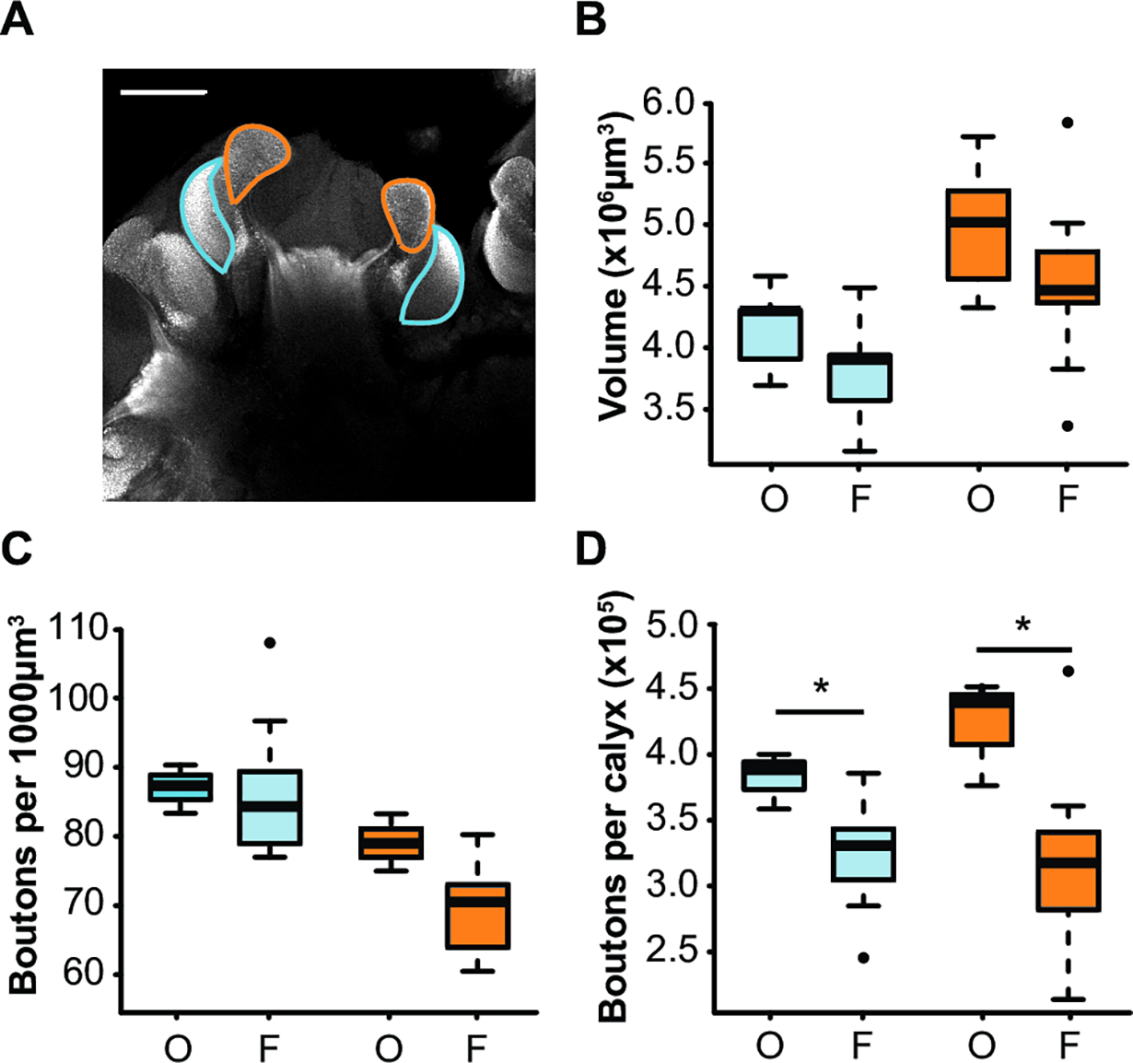
Mushroom body structure of orientating bees and foragers. **(A)** Frontal confocal image of the right median MB labelled for synapsin (scale bar = 100μm). Borders of the lip (*orange*) and dense collar (*blue*) are highlighted. Boxplots showing the characteristics of the dense collar (*blue*) and lip (*orange*) of a sample of orientating bees (*O*, n = 5) and foragers (*F*, n = 13): **(B)** neuropil volume, **(C)** density of synaptic boutons, **(D)** number of synaptic boutons per neuropil. * p < 0.05, Mann-Whitney U-Test.

Mushroom body structure was compared between orientating bees and foragers, regardless of their foraging duration **(Fig 4A)**. The volumes of the lip and dense collar did not differ significantly between orientating bees and foragers (Mann-Whitney U-test: *lip*: U = 18, p = 0.173; *collar*: U = 16, p = 0.117) **(Fig 4B)**, and neither did synaptic bouton density (*lip*: U = 5, p = 0.060; *dense collar*: U = 15.5, p = 0.638) **(Fig 4C)**. However, the extrapolated total number of synaptic boutons in the lip and dense collar was lower in foragers than in orientating bees (*lip*: U = 3, p < 0.05; *collar*: U = 3, p < 0.05) **(Fig 4D)**, indicating that the transition from orientation flights to foraging was likely accompanied by an overall decrease in synaptic bouton number in both regions.

**Fig 5:**
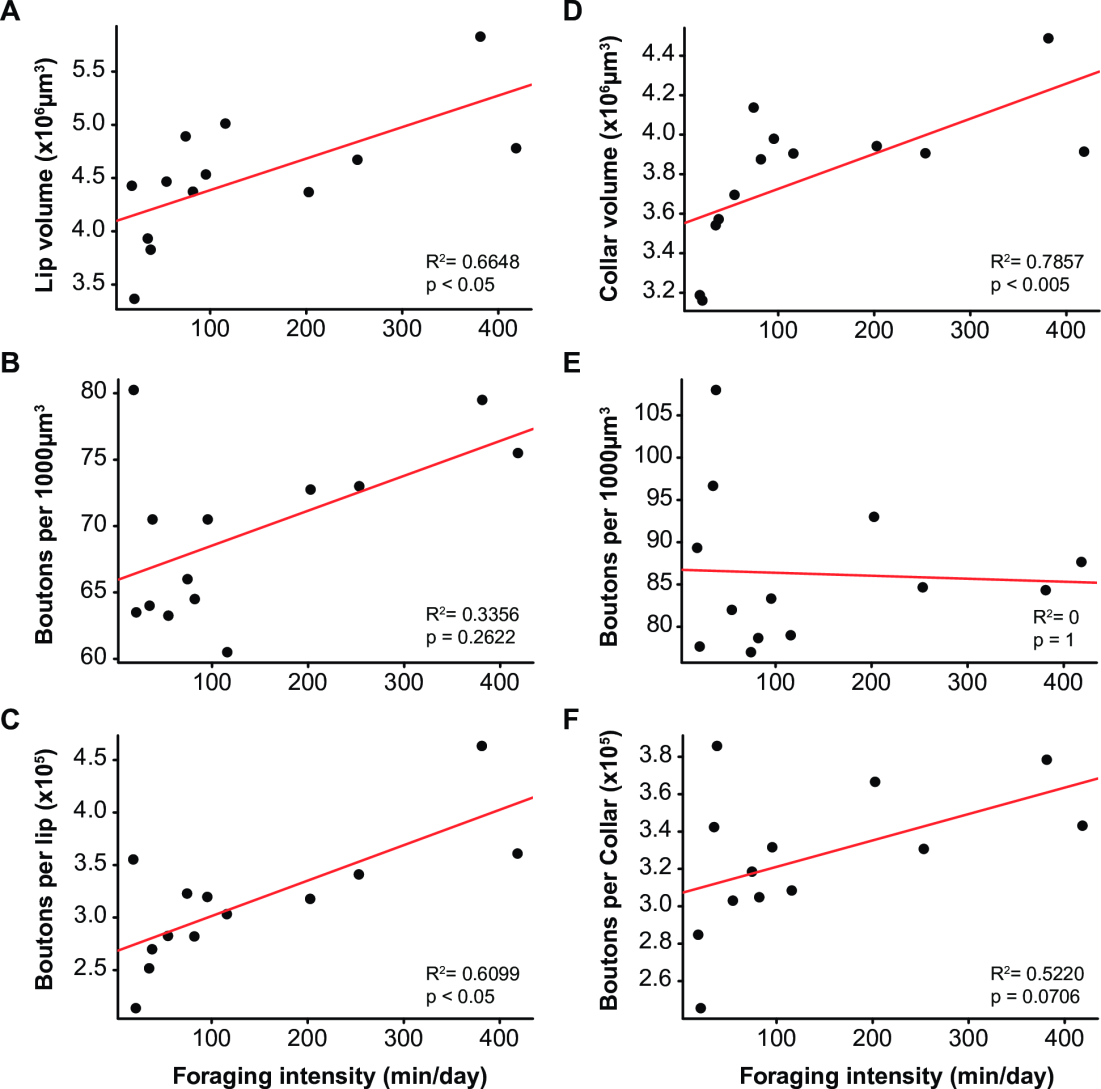
Correlations between foraging intensity and structural characteristics of the mushroom bodies. Individual values (n = 13) for the parameters of the lip **(A, B, C)** and dense collar **(D, E, F)** are plotted against foraging intensity: neuropilar volume **(A, D)**, density of synaptic boutons **(B, E),** total number of synaptic boutons **(C, F)**. The volume of the lip and collar, as well as the total number of boutons per lip, correlate positively with foraging intensity (Spearman rank correlations).

Because the sampled foragers included mostly foragers with short foraging durations, we could not assess whether MB structure varied with foraging duration. Thus, we considered foraging intensity (calculated as foraging duration/foraging day), which varied more between individuals **(Fig 5)**. Foraging intensity was positively correlated with the volume of the lip and dense collar (Spearman’s rank correlation; *lip*: R^2^ = 0.665, p < 0.05; *collar*: R^2^ = 0.786, p < 0.005), and with the total number of synaptic boutons in both regions (*lip*: R^2^ = 0.610, p < 0.05; *collar*: R^2^ = 0.522, p = 0.071). Intense foraging was associated with a larger MB neuropil containing a higher number of synaptic boutons. Foraging intensity was also related to performances in the 4^th^ trial of the reversal phase, as non-learners had a higher foraging intensity than learners (Trial 4: U = 888, p < 0.05; Trial 5: U = 966, p = 0.162) **(S1 Fig)**.

### Success in reversal learning is associated with a low number of synaptic boutons in the MBs

**Fig 6:**
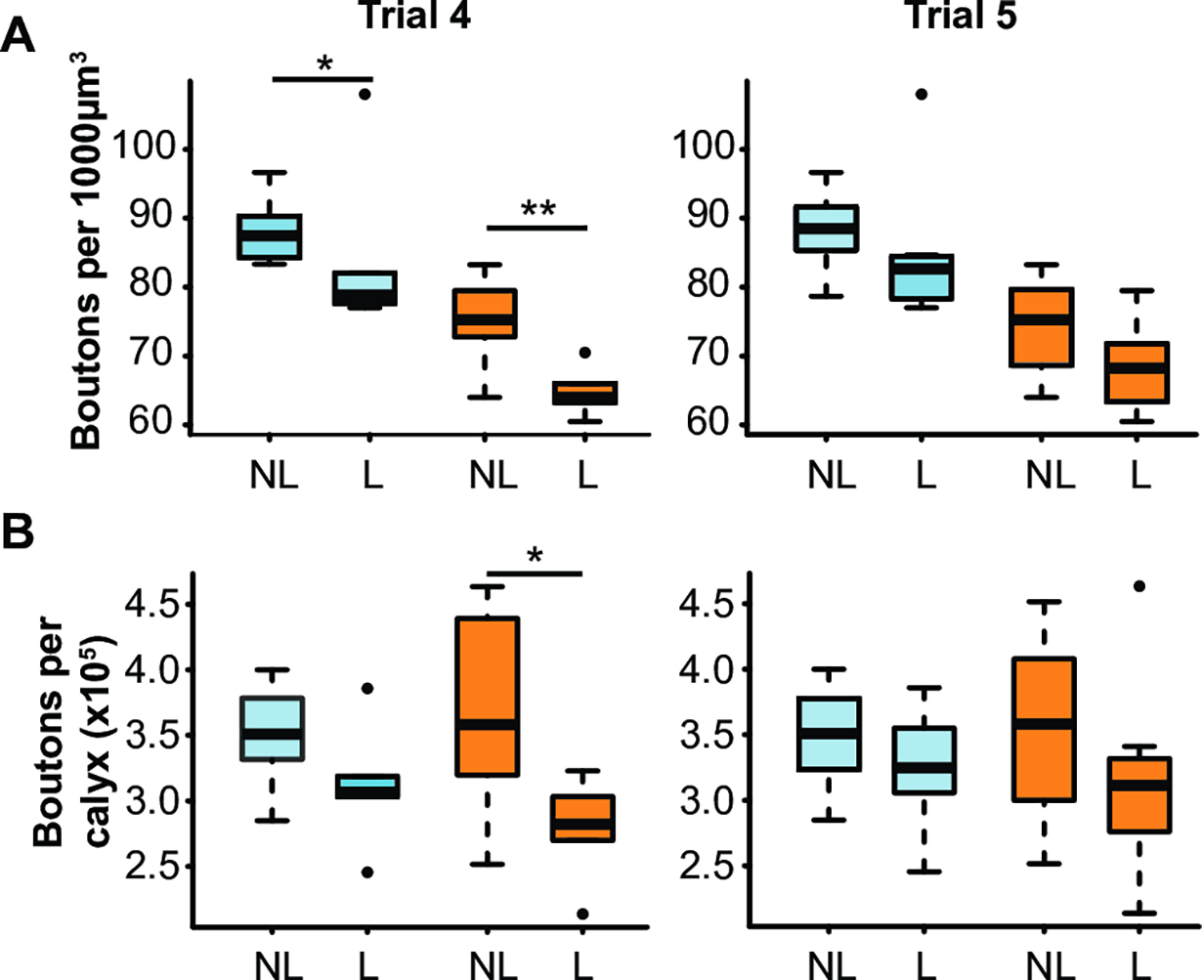
Synaptic bouton density and number and reversal learning performance. Boxplots showing the characteristics of the dense collar (*blue*) and lip (*orange*) of non-learners (*NL*, IS = -1 or 0) and learners (*L*, IS = 1) for each of the last two trials of the reversal phase: **(A)** density of synaptic boutons, **(B)** number of synaptic boutons per neuropil. [*Trial 4*: n = 12 NL and 6 L; *Trial 5*: n = 10 NL and 8 L] * p < 0.05, ** p < 0.005, Mann-Whitney U-Test.

Finally, we compared the MB structure of bees that successfully reversed their learning in the last two trials of the reversal phase (learners) and bees that did not (non-learners), regardless of foraging intensity **(Fig 6)**. Variations in reversal learning performance were not associated with volume differences in either neuropil **(S2 Fig)**. Yet, synaptic boutons in both regions were less dense in learners than in non-learners in the 4^th^, but not in the 5^th^ trial **(Fig 6A)** (Trial 4: *lip*: U = 56.5, p < 0.005; *collar*: U = 50, p < 0.05; Trial 5: *lip*: U = 49, p = 0.083; *collar*: U = 48.5, p = 0.093). As a result, the total number of synaptic boutons in the lip and dense collar was lower in learners than in non-learners in the 4^th^ trial of the reversal phase **(Fig 6B)** (*Trial 4*: lip: U = 53, p < 0.05, collar: U = 48, p = 0.056; *Trial5*: lip: U = 42, p = 0.328, collar: U = 43, p = 0.279). These results suggest that a fast acquisition of reversal by the 4^th^ trial of the reversal phase was associated with fewer synaptic boutons in the MBs.

**Fig 7:**
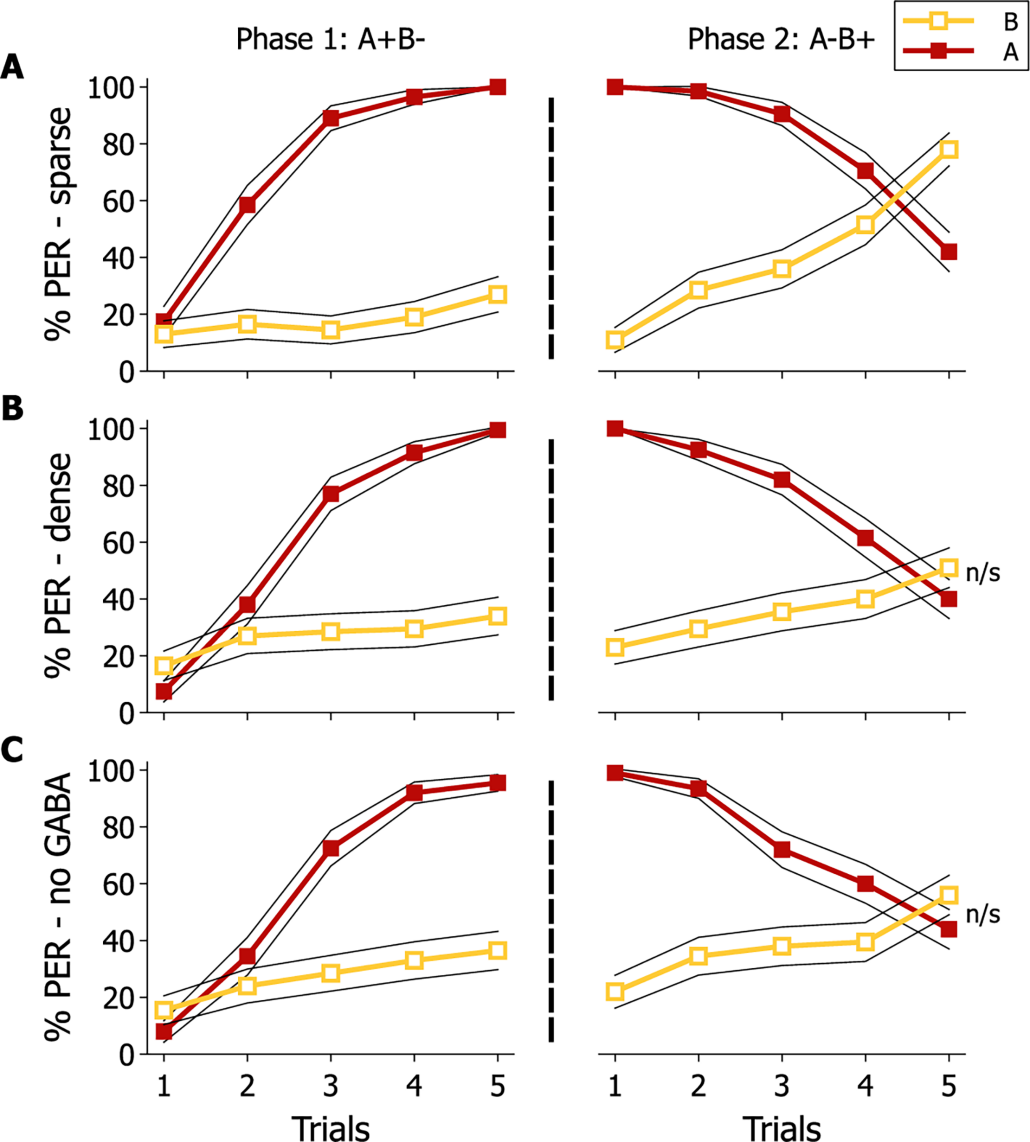
Modelled consequences of varying MB connectivity on reversal learning performance. Modelled percentage of individuals displaying PER in response to odours A (*red line*) and B (*orange line*) during the reversal learning paradigm. Three different models were run simulating a sparse **(A)** or dense **(B)** distribution of excitatory connections onto MB neurons (KC), and **(C)** sparse with suppressed inhibitory input from the GABAergic PCT. 200 agents (virtual bees) were modelled for each model configuration. The 95% confidence intervals are represented by the black lines.

In order to better understand the relationship between MB neural architecture and reversal learning, we used our model of the MBs to explore possible consequences of changing the connectivity between input neurons and MB neurons for reversal performance **(Fig 7)**. In the model, decreasing sparseness by increasing the number of input connections onto MB neurons impaired reversal learning despite efficient learning in the first phase. Removing inhibitory feedback from the GABAergic PCT neurons onto MB neurons also reduced reversal learning performance, thus showing the model was able to generate results similar to those reported in a prior experimental study [37]. The increase in sparseness decreases the number of Kenyon cells that respond to both stimuli, thus allowing faster changes in response to the stimuli as the changes to the weights due to learning only affect the two stimuli individually.

## Discussion

This study reports a clear, but complex relationship between experience-dependent plasticity in honey bee MB structure and variation in cognitive capacity. We show that a reduced number of synaptic boutons in the MB neuropil following orientation flights is associated with improved performance in reversal learning **(Figs 3 and 4)**. As bees accumulate more time foraging, however, synaptic bouton numbers increase **(Fig 5)** while reversal learning performance decreases **(Fig 1)**. Because of the precise measures of foraging experience provided by our RFID data we can here report a biphasic response of MB plasticity to foraging with an initial pruning of synaptic boutons at foraging onset followed by an increase in synaptic bouton number with more foraging **(Fig 1)**. Interestingly, changes in the volume of the MBs did not affected reversal learning performance. Volumetric changes of the MBs with foraging experience have been shown to reflect dendritic branching [18] and do not provide a precise measure of the synaptic connectivity therein: new synapses can be formed on pre-existing boutons or on new boutons. Our data are therefore consistent with previous reports of experience-dependent plasticity in bees [18,19,25], but illustrate more sophistication than has been previously recognised. Replicating such an experiment by using different learning tasks would test the strength of the reported relationship between foraging experience and synaptic bouton number in the MBs and would reveal whether synaptic bouton number influences other cognitive capacities in honey bees.

### Negative relationship between synaptic bouton number in the MBs and reversal learning performance

The synaptic pruning observed at foraging onset might be a consequence of the drastic change in environment and activity concomitant with orientation flights and the onset of foraging. Indeed, synaptic pruning has been reported previously in the dense collar of bees and ants following exposure to light [29,30]. Also, exposure to a rich olfactory environment was demonstrated to reduce synaptic bouton number in the lip of leaf-cutting ants [16]. The improved reversal learning performance of new foragers in our study suggests that this synaptic pruning is part of an optimisation of MB synaptic connectivity such that a lower synaptic bouton number in the calyx yields improved reversal learning performance.

Several authors have argued that for mammals synaptic pruning is an essential aspect of memory formation to optimise the differentiation of memory engrams [4,17,45]. It is typically assumed that there is a relationship between synapse number and the coding of information in neural circuits [46,47]. Synaptic pruning has been proposed to participate into the establishment of neuronal input selectivity which, in terms of sensory coding, would contribute to sparsening stimulus representations by reducing overlap between them [48]. In addition, sparse coding of information has been shown to be maintained by GABAergic inhibition of circuit connection strengths, which is needed for complex discriminations like reversal learning [49,50]. Consistently, studies in fruit flies and honey bees have shown that sparse coding of KC responses to odorants in the lip is necessary to discriminate between similar odours [42], and that GABAergic input to the MBs (presumably from feedback PCT/A3 neurons), which contributes to sparse coding of olfactory representation in the lip [41,51], is required to solve a reversal learning task [37,52]. In our model of the MBs, removing the inhibitory GABAergic input to the MBs also reduced reversal learning performance, suggesting the model is effectively capturing the biology of the MBs as an odour learning system.

In the model we could adjust the density of connections between the IN and KC and thereby alter the degree of sparseness of odour coding. Doing so had little effect on the acquisition of odour learning in the first phase of training but had a significant impact on the reversal of odour learning. This was because for a reversal of learning to occur, the net weights connection strengths at the output of the MBs had to change such that there was a reversal in whether the Extend or Retract subpopulations of the EN were activated more strongly. Such a reorganisation of relative connection strengths at the MB output occurred more quickly in our model when connection density was low (sparse coding of information) than when connection density was high. The model analyses presented a match to our experimental data and our model suggests a possible explanation for why a lower number of synaptic boutons correlates with improved reversal learning performance.

We recognise, however, that synaptic boutons are complex of synapses rather than single synapses [38,53]. With the resolution of our microsocopy studies, we cannot rule out that foraging experience does not affect the overall number of *synapses*, even if that of boutons is reduced. Age-related synaptic pruning has been associated with increased number of post-synaptic partners per bouton, and a change in the proportion of different synaptic types (non-ribbon *vs*. ribbon synapses), as observed under electron microscopy [38]. The value of such ultrastructural parameters as proxies for synaptic strength remains unclear because the functional status of different synaptic types remains unclear (38). Whether these changes in synapse number within existing boutons represent additional contacts with already-connected KC (thereby having no effect on sparse coding) or with a greater number of KC (thereby potentially changing sparseness of coding of odours) is unknown. Here we argue simply that if we can assume a relationship between synaptic bouton number and the density of coding of an odour signal within the KC population, then our model suggests a mechanistic explanation for why lower synaptic bouton number was associated with better reversal learning performance.

We observed similar experience-dependent changes in synaptic boutons in both the collar and the lip of the MBs. The collar is a visual input region, and future studies should investigate the link between synaptic bouton number in the collar and visual reversal learning as presently the relationship between synaptic bouton number and learning of visual information is unclear. In fruit flies, visual reversal learning has also been shown to be improved by GABAergic inhibition [54], suggesting that sparse coding might be beneficial to solve this task. In honey bees, the density of synaptic boutons in the collar was not related to performance in a 2-colour discrimination task [55], but bumblebees with a high density of synaptic boutons in the collar have been shown to learn faster to discriminate between 10 different colours [56]. A possibility might be that increasing number of synaptic boutons in honey bees with greater experience might facilitate some learning tasks (not tested here) to the expense of others (such as reversal learning). Clearly more work is needed on visual learning to reconcile these findings.

### Variation in reversal learning performance and synaptic bouton number in forager bees

Our data show that reversal learning performance was highest in young foragers but declined as bees accumulated foraging experience. The reversal learning task we used assays how effectively bees could update an existing learned association with new information. This capacity would be vital for a forager honey bee because the availability of nectar and pollen in the environment changes rapidly both between and within flower types [57,58]. It is telling that our data suggest MB microstructure is optimised for updating newly learned information when a bee starts to forage, with the consequence being a new forager could rapidly adjust foraging preferences to track a changing availability of floral resources in the environment. With increasing foraging experience, this flexibility in learned behaviours decreased.

The decreased reversal learning performance in our study might be part of a general cognitive decline as consequence of foraging effort, as has been suggested by previous studies. Compared to in-hive workers or young foragers, experienced foragers have been shown to perform poorly in an absolute olfactory learning task [59,60], a tactile learning task [61], and a spatial memory extinction task [62]. By comparing same-age foragers, we suggest that foraging activity itself may be deleterious for cognitive capacities in honey bees. Alternatively, the decreased flexibility in experienced-foragers may also be interpreted as adaptive at a colony-level. One may argue that efficient foraging requires some bees to be more persistent than others in foraging on a floral species or patch. A pool of bees with differing amount of flexibility in foraging choices has been shown to be beneficial for the colony [32].

What might have caused the increased synaptic bouton number in experienced foragers? In mammals it is argued that functional optimisation of circuit connectivity for effective learning is dependent on a fine balance between excitation and inhibition in the neural circuit [3,63]. We argue similar principles likely apply to bees. We have already discussed how reversal learning performance is dependent on GABAergic inhibition of the MBs [37,52]. Excitation of the MBs by excitatory cholinergic neurotransmission is higher in foragers than in nurses [31] and the increase in MB volume and dendritic arborisation observed in foragers can be triggered by a chronic stimulation of the muscarinic receptors to acetylcholine [19,20]. We propose a possible shift in the excitation/inhibition balance in the MBs with intense foraging resulting in a suboptimal increase in synaptic boutons.

With this perspective it is interesting that the decrease in reversal learning performance with foraging experience was more apparent in precocious foragers than in normal-age foragers. Precocious foraging results from a stress applied to the colony, such as depleting a part of the foraging force, nutritional stress, disease stress or pesticide exposure [23,33,36]. Precocious foragers perform less well than normal-age foragers in a range of foraging related metrics [33,34,64]. The susceptibility of precocious foragers to foraging-related decline in reversal performance is reminiscent of examples from the mammal literature linking stress to imbalances in brain neurochemistry and reduced cognitive performance [50,65,66]. Importantly, this might also explain why precocious foragers perform so poorly as foragers in the field [34]. Yet, further studies should investigate specifically the impact of stressors on MB neuronal circuitry and learning performance.

### Conclusion

Here, we show how experience-dependent variation in brain microstructure relates to individual variation in cognitive performance. We argue the mechanistic explanation for the relationship is an optimisation of synaptic bouton number for effective memory storage, which is achieved by a fine balance of excitation and inhibition in neural circuits. We suggest these principles for brain and behavioural plasticity operate similarly in all animals. Our study has highlighted the value of examining neuroplasticity within the natural and ecological context of the animal, and of considering inter-individual variation in brain structure and behaviour as signal rather than noise [2].

## Supporting information

S1 Fig Foraging behaviour and reversal learning performance. Boxplots showing the foraging intensity (foraging duration/foraging day) of non-learners (*NL*, n = 61) and learners (*L*, n = 22) in the 4^th^ trial of the reversal phase. * p < 0.05, Mann-Whitney U-Test.
(TIF)

S2 Fig Mushroom bodies volume and reversal learning performance. Boxplots showing the volume of the dense collar (*blue*) and lip (*orange*) of non-learners (*NL*, IS = -1 or 0) and learners (*L*, IS = 1) for each of the last two trials of the reversal phase (*Trial 4*: n = 12 NL and 6 L; *Trial 5*: n = 10 NL and 8 L). Performance in reversal learning was not associated with differences in the volume of the lip and dense collar (Mann-Whitney U-Test; Trial 4: *lip*: U = 49, p = 0.2496; *collar*: U = 53, p = 0.1246; Trial 5: *lip*: U = 40, p = 1; *collar*: U = 44, p = 0.7618).
(TIF)
