# Supplementary figures and images for: Relationship between brain plasticity, learning and foraging performance in honey bees
Source: PLoS One. 2018 Apr 30;13(4):e0196749. doi: 10.1371/journal.pone.0196749 (PMC5927457; doi:10.1371/journal.pone.0196749)

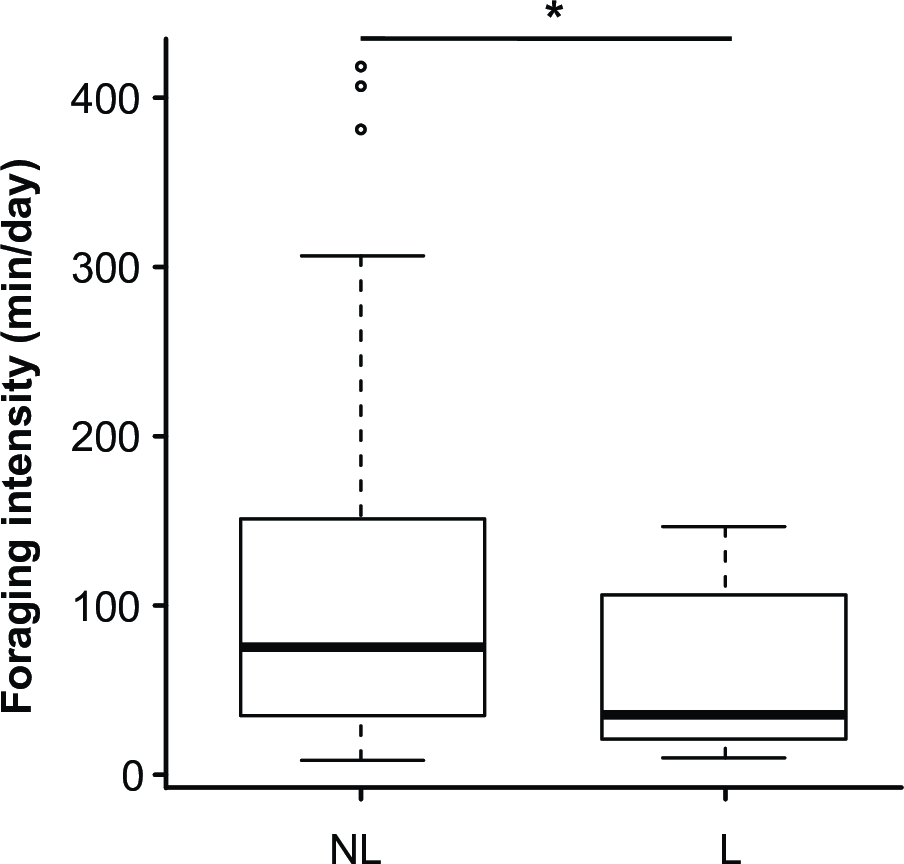

Supplement: S1 Fig — Boxplots showing the foraging intensity (foraging duration/foraging day) of non-learners (NL, n = 61) and learners (L, n = 22) in the 4th trial of the reversal phase. * p < 0.05, Mann-Whitney U-Test. (TIF) [file pone.0196749.s001.tif]

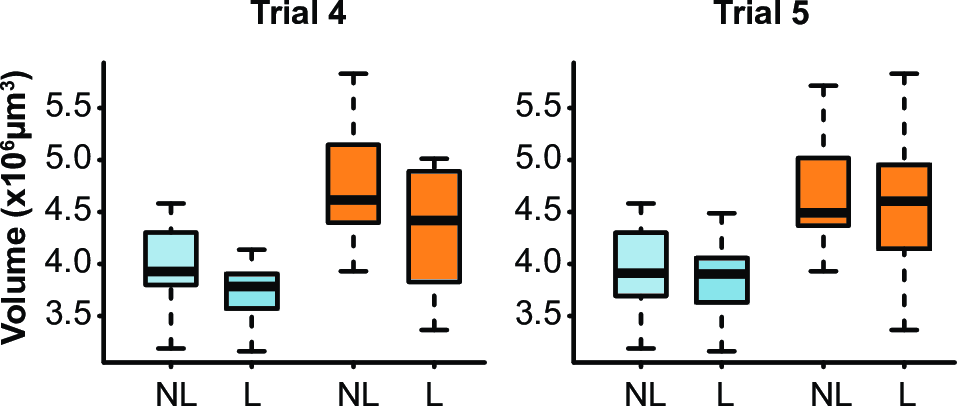

Supplement: S2 Fig — Boxplots showing the volume of the dense collar (blue) and lip (orange) of non-learners (NL, IS = -1 or 0) and learners (L, IS = 1) for each of the last two trials of the reversal phase (Trial 4: n = 12 NL and 6 L; Trial 5: n = 10 NL and 8 L). Performance in reversal learning was not associated with differences in the volume of the lip and dense collar (Mann-Whitney U-Test; Trial 4: lip: U = 49, p = 0.2496; collar: U = 53, p = 0.1246; Trial 5: lip: U = 40, p = 1; collar: U = 44, p = 0.7618). (TIF) [file pone.0196749.s002.tif]
